# Supplementary material for: Empirical methods for controlling false positives and estimating confidence in ChIP-Seq peaks
Source: BMC Bioinformatics. 2008 Dec 5;9:523. doi: 10.1186/1471-2105-9-523 (PMC2628906; doi:10.1186/1471-2105-9-523)
Supplement: Additional File 1 — A variety of html documents from the USeq web site detailing the available applications, their best usage, output file type descriptions, command line menus, etc. [file 1471-2105-9-523-S1.zip › USeqUserGuides/JavaDoc.zip!/JavaDoc/trans/main/WindowBlockMaker.html]

WindowBlockMaker


|  |  |  |
| --- | --- | --- |
| **Overview** \| **Package** \| **Class** \| **Use** \| **Tree** \| **Deprecated** \| **Index** \| **Help** | |  |
| **PREV CLASS**   **NEXT CLASS** | **FRAMES**    **NO FRAMES**     **All Classes** |  |
| SUMMARY: NESTED \| FIELD \| CONSTR \| METHOD | DETAIL: FIELD \| CONSTR \| METHOD |  |


---


## trans.main Class WindowBlockMaker

```
java.lang.Object
  trans.main.WindowBlockMaker
```

---

``` public class WindowBlockMaker extends java.lang.Object ```

Converts a Window[] into a heat map specific sgr file for import into IGB.
Assumes windows are sorted by chromosome and start position
Score index is the index to use when fetching a score for a window from it's double[] of scores
see ScanChip or ScanChromosomesCNV for current score indexes.

---

**Constructor Summary**

`WindowBlockMaker(int sizeOfOligo)`

`WindowBlockMaker(int sizeOfOligo,
java.lang.String genomeVersion)`

`WindowBlockMaker(java.lang.String[] args)`


| **Method Summary** | |
| --- | --- |
| `boolean` | `baseOverlapsWindow(int bp, Window win)`             Is a given base contained within the window? |
| `float` | `getBaseScore()` |
| `int` | `getScoreIndex()` |
| `double` | `highestScoringWindow(int base, int currentWindowIndex)`             Looks forward at overlapping windows, returns highest score for the given bp position. |
| `static void` | `main(java.lang.String[] args)` |
| `void` | `makeHeatMapBarFiles(Window[] windows, java.io.File directory)`             Be sure to set the scoreIndex, defaults to 0. |
| `void` | `makeHeatMapGrFiles(Window[] windows, java.io.File directory)`             Be sure to set the scoreIndex, defaults to 0. |
| `void` | `makeHeatMapSgrFile(Window[] windows, java.io.File file, boolean zipCompressIt)`             Be sure to set the scoreIndex, defaults to 0. |
| `static void` | `printDocs()` |
| `void` | `processArgs(java.lang.String[] args)`             This method will process each argument and assign new varibles |
| `void` | `setBaseScore(float baseScore)` |
| `void` | `setScoreIndex(int scoreIndex)` |

**Methods inherited from class java.lang.Object**

`equals, getClass, hashCode, notify, notifyAll, toString, wait, wait, wait`

**Constructor Detail**

### WindowBlockMaker

```
public WindowBlockMaker(int sizeOfOligo)
```

---


### WindowBlockMaker

```
public WindowBlockMaker(int sizeOfOligo,
                        java.lang.String genomeVersion)
```

---


### WindowBlockMaker

```
public WindowBlockMaker(java.lang.String[] args)
```


**Method Detail**

### makeHeatMapGrFiles

```
public void makeHeatMapGrFiles(Window[] windows,
                               java.io.File directory)
```

:   Be sure to set the scoreIndex, defaults to 0. Automatically zips them

---


### makeHeatMapSgrFile

```
public void makeHeatMapSgrFile(Window[] windows,
                               java.io.File file,
                               boolean zipCompressIt)
```

:   Be sure to set the scoreIndex, defaults to 0.

---


### makeHeatMapBarFiles

```
public void makeHeatMapBarFiles(Window[] windows,
                                java.io.File directory)
```

:   Be sure to set the scoreIndex, defaults to 0. Automatically zips them

---


### highestScoringWindow

```
public double highestScoringWindow(int base,
                                   int currentWindowIndex)
```

:   Looks forward at overlapping windows, returns highest score for the given bp position.

---


### baseOverlapsWindow

```
public boolean baseOverlapsWindow(int bp,
                                  Window win)
```

:   Is a given base contained within the window?

---


### main

```
public static void main(java.lang.String[] args)
```

---


### processArgs

```
public void processArgs(java.lang.String[] args)
```

:   This method will process each argument and assign new varibles

---


### printDocs

```
public static void printDocs()
```

---


### getScoreIndex

```
public int getScoreIndex()
```

---


### setScoreIndex

```
public void setScoreIndex(int scoreIndex)
```

---


### getBaseScore

```
public float getBaseScore()
```

---


### setBaseScore

```
public void setBaseScore(float baseScore)
```


---


|  |  |  |
| --- | --- | --- |
| **Overview** \| **Package** \| **Class** \| **Use** \| **Tree** \| **Deprecated** \| **Index** \| **Help** | |  |
| **PREV CLASS**   **NEXT CLASS** | **FRAMES**    **NO FRAMES**     **All Classes** |  |
| SUMMARY: NESTED \| FIELD \| CONSTR \| METHOD | DETAIL: FIELD \| CONSTR \| METHOD |  |


---
